# Network‐based method for detecting dysregulated pathways in glioblastoma cancer

**DOI:** 10.1049/iet-syb.2017.0033

**Published:** 2018-02-01

**Authors:** Hao Wu, Jihua Dong, Jicheng Wei

**Affiliations:** ^1^ College of Information Engineering Northwest A&F University Yangling 712100 Shaanxi People's Republic of China; ^2^ Department of Foreign Languages Northwest A&F University Yangling 712100 Shaanxi People's Republic of China

**Keywords:** cancer, tumours, drugs, brain, neurophysiology, genetic algorithms, genetics, skin, proteins, molecular biophysics, genomics, patient diagnosis, molecular configurations, network‐based method, dysregulated pathways detection, glioblastoma cancer, biological molecular mechanisms, precise diagnosis, cancer patient treatment, drug targets, mutual exclusivity, mutated genes, gene mutations, expression changes, expression data, CFinder clustering algorithm, constructed gene network, gene sets, overlapping scores, glioblastoma‐related multiple genes, epidermal growth factor receptor, TP53, secondary subtype

## Abstract

The knowledge on the biological molecular mechanisms underlying cancer is important for the precise diagnosis and treatment of cancer patients. Detecting dysregulated pathways in cancer can provide insights into the mechanism of cancer and help to detect novel drug targets. Based on the wide existing mutual exclusivity among mutated genes and the interrelationship between gene mutations and expression changes, this study presents a network‐based method to detect the dysregulated pathways from gene mutations and expression data of the glioblastoma cancer. First, the authors construct a gene network based on mutual exclusivity between each pair of genes and the interaction between gene mutations and expression changes. Then they detect all complete subgraphs using CFinder clustering algorithm in the constructed gene network. Next, the two gene sets whose overlapping scores are above a specific threshold are merged. Finally, they obtain two dysregulated pathways in which there are glioblastoma‐related multiple genes which are closely related to the two subtypes of glioblastoma. The results show that one dysregulated pathway revolving around epidermal growth factor receptor is likely to be associated with the primary subtype of glioblastoma, and the other dysregulated pathway revolving around TP53 is likely to be associated with the secondary subtype of glioblastoma.

## 1 Introduction

Cancer has become one of the most serious threats to human health. It is driven by a variety of factors, including multiple gene mutations and a variety of biological process disorders, which make the process of cancer development much more complex [1]. Currently, the methods for detecting cancer biomarkers and therapeutic targets mainly focus on analysing genetic, transcriptomic, proteomic and epigenetic data [2]. One of the greatest challenges in biomarker discovery is the heterogeneity of the same type of cancer, while at the same time, gene mutations are highly heterogeneous and vary from one sample to another. Therefore, a pattern identified in one study is often found unsuccessful in different data sets [3]. Clinically, this heterogeneity is mainly due to the following reasons. First, clinical data come from different platforms and protocols. Second, the existing cancer data sets are usually cross‐sectional biomolecules. This means that the data sets coming from the same time point measurements for all patients may not necessarily be the informative ones. Third, the inaccurate clinical diagnosis may lead to incorrect assessment of clinical samples [3].

Previous studies have detected differential expression genes between normal samples and cancer samples, and found that they are closely related to cancer [2, 4]. It has also been found that many genes detected by the differential expression method in a data set often do not work in another [4]. The cause of this phenomenon may be related to prior hypothesis. Although differential expression genes are associated with cancer, the development of cancer is usually caused by a dysregulated pathway of a group of genes [2–4].

Additionally, different patients with the same type of cancer have different genetic abnormalities, but at least one subset of these abnormalities is consistent in a group of patients [4]. This suggests that several combinations of gene mutations and patterns of expression may lead to similar changes in cancer cell apoptosis, mutation and proliferation [5]. That is, different biomolecules may lead to the growth and spread of cancer cells in a similar manner.

Currently, there are a large number of high‐throughput data and human genome sequencing data available, such as gene expression, gene mutations and protein–protein interaction data, which provide ample data support for detecting pathogenic genes and dysregulated pathways in cancer [1, 2, 4, 6–9]. Based on these data sets, the following two computational approaches have been widely used to detect mutually exclusive pathogenic functional modules. The first approach is to directly identify driver pathways de novo from somatic mutation data utilising two combinatorial properties, namely high coverage and high exclusivity, without integrating any additional biological information and prior knowledge [5–7]. The second is to detect cancer‐related dysregulated pathways utilising the mutual exclusivity of mutated genes and the gene co‐expression principle in gene expression data. Drawing upon these approaches, researchers have detected cancer‐related dysregulated modules by integrating gene mutations and gene expression data [1, 10, 11].

Recently, researchers have analysed the genome spectrum to study how mutated genes occur in one pathway, and proposed two combinatorial properties, namely high exclusivity and high coverage [6]. Most biologists now believe that mutually exclusive mutated gene sets provide strong evidence for the association between functional mutations and biological pathways [12, 13]. Accordingly, many computational approaches have been proposed to detect driver pathways and dysregulated pathways in cancer based on the two combinatorial properties.

For instance, Vandin *et al.* [7] proposed the de novo driver exclusivity (Dendrix) algorithm to detect mutated driver pathways from gene mutations data in cancer. In order to obtain high exclusive gene sets, the study introduces the weight function $W\left(M\right)=\left|\Gamma (M)\right|-\omega \left(M\right)=2\left|\Gamma \left(M\right)\right|-\sum_{g\in M}\left|\Gamma (g)\right|$ to penalise the overlap between genes and reward the coverage. The study presents a straightforward greedy algorithm and a Markov Chain Monte‐Carlo approach to find gene sets with high coverage and exclusivity. The algorithm computes the weight fractions of each gene set, and selects the set of genes with the highest weight as a mutated driver pathway, then removes the nodes and repeats the above iteration until finds all gene sets which meet high coverage and exclusivity. Although this approach can identify the gene sets with the highest weight, this iterative method only produces a locally optimal solution, and requires designating the number of genes in a driver pathway in advance. Leiserson *et al.* [5] improved the Dendrix algorithm and proposed a multi‐Dendrix algorithm that can simultaneously detect multiple driver pathways by treating the maximum weight subarray as a linear programming problem, defining the objective function and setting the constraint condition to find the maximum weight subarray problem that satisfies the condition.

Zhao *et al.* [10] proposed mutated driver pathway finder (MDPFinder) algorithm to detect driver pathways by integrating gene mutation and gene expression data. The study hypothesises that the genes in the same pathway are inclined to perform the same biological function, and they are usually regarded to have higher correlation than those in different pathways. In the study, they firstly filter out the genes with high mutual exclusivity but low correlation according to the above principle, and then define a new function $F_{ME}=W\left(M\right)+\lambda \times R(E_{M})$, where $W\left(M\right)$ is the scoring function used to penalise the overlap between genes and to reward the coverage, $E_{M}$ is the gene expression matrix, which has the same gene set as the mutation matrix ***M***. To determine the optimal boundary ranges of scoring functions, they proposed a genetic algorithm model to solve the so‐called maximum weight submatrix problem.

Kim *et al.* [12] proposed MEMCover algorithm to detect dysregulated modules utilising somatic mutation data in cancer. In the algorithm, the relationship between genes is determined based on mutual exclusivity between a pair of mutated genes. This algorithm classifies mutual exclusivity into three categories: mutual exclusivity within a cancer type, mutual exclusivity between cancer types and mutual exclusivity across multiple cancer types. By combining tissue specificity and commonness, the algorithm detects dysregulated modules containing rare mutation genes which exist in the process of cancer development and progression.

Different sets of mutated genes may cause different subtypes of cancer, so detecting the mutated gene sets in cancer is useful for studying cancer pathogenesis and drug targets. Gene mutations may be caused by other gene expression changes, and gene expression changes may also be caused by other gene mutations. Therefore, integrating gene mutations and expression data to detect cancer‐related dysregulation pathways helps us to identify cancer mechanisms and drug targets [3]. This study proposes a network‐based method to identify dysregulated pathways utilising mutual exclusivity between mutated genes and the interrelationship between gene mutations and gene expression changes by integrating gene mutations and expression data in cancer.

## 2 Methods

### 2.1 Integrated method between gene mutations and expression data

The method to construct the relationship between gene mutations and expression changes is presented below. We introduce m×n binary mutation matrix ***A*** and expression matrix ***E***, where *m* represents the number of patient samples and *n* represents the number of mutated genes in the data set. In mutation matrix ***A***, $a_{ij}=1$ indicates that gene $g_{j}$ mutates in the sample $s_{i}$, otherwise $a_{ij}=0$. Element $e_{ij}$ in the gene expression matrix refers to the expression level of gene $g_{j}$ in the sample $s_{i}$. Also, the *connectivity* between expression gene $g_{k}$ and mutation gene $g_{h}$ is defined as the product between the expression level of gene $g_{k}$ and the mutation status of gene $g_{h}$. Hence, we build the *connectivity* matrix $CAE=A^{T}E$ to represent gene mutations effect on expression changes of other genes. We construct the second *connectivity* matrix $CEA=E^{T}A$ to represent the effect of gene expression changes on other gene mutations. The value of entries $CAE_{hk}$ and $CEA_{kh}$ of the *connection* matrix can be interpreted as follows: if the values of $CAE_{hk}$ or $CEA_{kh}$ are closer to zero, most samples exhibit small absolute values of the expression level for the gene $g_{k}$ or most samples have no mutation in gene $g_{h}$; conversely, if the values of $CAE_{hk}$ and $CEA_{kh}$ are far from zero, there is a strong correlation between the *connectivity* of gene $g_{h}$ and gene $g_{k\;}$ across the data set [3]. It is thus clear that a *connectivity* is obtained when $\left|CAE_{hk}\right|/\text{num}(a_{ih})\geq \sum_{i=1}^{m}\left|e_{ik}\right|/m\;$ or $\left|CEA_{kh}\right|/\text{num}(a_{ih})\geq \sum_{i=1}^{m}\left|e_{ik}\right|/m$ for i=1,2,…,m and ∀ pair ($g_{h}$, $g_{k}$) of mutation and expression genes, where $\text{num}\left(a_{ih}\right)$ represents the number of gene $g_{h}$ mutation in all samples. Note that gene pair ($g_{h}$, $g_{k}$) has a strong connectivity score when gene $g_{h}$ mutates and gene $g_{k}$ is most under/over expressed in the same samples.

### 2.2 Exclusivity and weight

Mutual exclusivity in mutated genes widely exists in cancer genomes. The genes in one exclusive gene set usually have at most one gene mutation in one patient, and perform the same or similar functions. Therefore, identifying these mutually exclusive sets of genes is a crucial step in studying the pathogenesis of cancer.

**Fig. 1:**
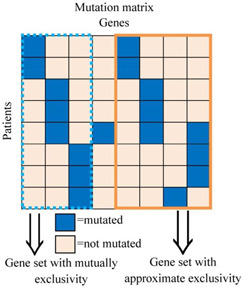
Mutation matrix in multiple patients

For gene *g* in the mutation matrix ***A***, the coverage function $\Gamma \left(g\right)=\{i|a_{ij}=1\}$ represents the set of patients in which gene $g_{j}$ mutates (Fig. 1). For $g_{i},g_{j}\in A,g_{i}\neq g_{j},1\leq i,j\leq n$, $\Gamma (g_{i})\cap \Gamma (g_{j})=\emptyset$ shows genes $g_{i}$ and $g_{j}$ are mutually exclusive. The degree of exclusivity, coverage and weight of the two genes $g_{i}$ and $g_{j}$ are defined as follows.

For a pair of genes $g_{i}$ and $g_{j}$ in the mutation matrix ***A***, the exclusive degree function $\text{ED}(g_{i},g_{j})$ is defined as

(1) $$\text{ED}(g_{i},g_{j})=\frac{\left|\Gamma (g_{i})\cup \Gamma (g_{j})\right|}{|\Gamma (g_{i})|+\left|\Gamma (g_{j})\right|}$$

According to the above analysis, $\text{ED}(g_{i},g_{j})=1$, when the pair of genes $g_{i}$ and $g_{j}$ are mutually exclusive.

For a pair of genes $g_{i}\;$ and $g_{j}$ in the mutation matrix ***A***, the coverage degree function $\text{CD}(g_{i},g_{j})$ is defined as

(2) $$\text{CD}(g_{i},g_{j})=\frac{\left|\Gamma (g_{i})\cup \Gamma (g_{j})\right|}{m}$$

Considering both the coverage overlap and gene coverages $\Gamma (g_{i})$ and $\Gamma (g_{j})$, the weight degree function $\text{WD}\left(g_{i},g_{j}\right)$ is defined as

(3) $$\text{WD}\left(g_{i},g_{j}\right)=1-\frac{\left|\Gamma (g_{i})\cap \Gamma (g_{j})\right|}{\min\left\{\;\left|\Gamma (g_{i})\right|,\left|\Gamma (g_{j})\right|\right\}}$$

**Fig. 2:**
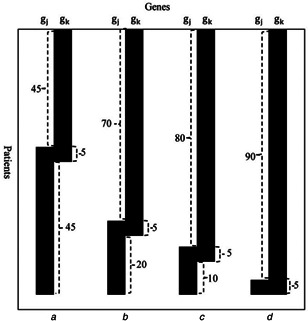
Weight degree analysis of two genes with the same coverage, coverage overlap and exclusive degree

For mutated genes $g_{i}$ and $g_{j}$, if the coverage of one gene is low and the coverage of the other gene is very high, then the exclusive degree $\text{ED}(g_{i},g_{j})$ of the two genes is high but it does not mean that the weight degree $\text{WD}(g_{i},g_{j})$ of the two genes is high (i.e. Figs. 2*c* and *d*). Therefore, many pairs of genes may be filtered by the weight degree function $\text{WD}(g_{i},g_{j})$.

### 2.3 Constructing a gene network based on connectivity and exclusivity

Based on the integrated model of the gene mutations and expression data discussed above, a gene network is constructed using mutual exclusivity between genes and the interaction between gene mutations and expression changes. A set of genes that are mutually exclusive or that interact with each other in their mutations and expression changes may have a major impact on cancer [14, 15]. Hence, formula (1) is used to calculate the exclusive degree between any pair of genes, and formula (3) is used to calculate weight degree between the pair of genes. If $\text{ED}(g_{h},g_{k})\geq \lambda$ and $\text{WD}\left(g_{h},g_{k}\right)\geq \gamma$ or $\left|CAE_{hk}\right|/\text{num}(a_{ih})\geq \sum_{i=1}^{m}\left|e_{ik}\right|/m$ or $\left|CEA_{kh}\right|/\text{num}(a_{ih})\geq \sum_{i=1}^{m\;}\left|e_{ik}\right|/m$, then an edge is created to link the nodes $g_{h}$ and $g_{k}$. and then we construct a gene network in which each node represents a gene and each edge represents the two connected genes that maintain a mutually exclusive relationship or interrelationship between a gene mutation and a gene expression change. If one gene is not connected to any other genes, the gene does not appear in the network. The process of constructing a gene network is described as follows:

*Step 1:* The gene network *G* is initialised to a zero matrix.
*Step 2:* Calculate the effect of gene mutations on gene expression changes $CAE=A^{T}E$.
*Step 3:* For the mutated gene $g_{h}$ and the expression gene $g_{k}$, if $\left|CAE_{hk}\right|/\text{num}(a_{ih})\geq \sum_{i=1}^{m}\left|e_{ik}\right|/m$, then G[h][k]=1.
*Step 4:* Calculate the effect of gene expression changes on gene mutations $CEA=E^{T}A$.
*Step 5:* For the expression gene $g_{h}$ and the mutated gene $g_{k}$, if $\left|CEA_{kh}\right|/\text{num}(a_{ih})\geq \sum_{i=1}^{m}\left|e_{ik}\right|/m$ Then G[h][k]=1.
*Step 6:* For the mutated genes $g_{h}$ and $g_{k}$, if $\text{ED}(g_{h},g_{k})\geq \lambda$ and $\text{WD}\left(g_{h},g_{k}\right)\geq \gamma$ then G[h][k]=1.
*Step 7:* Gene network *G* is constructed, and the process ends.

### 2.4 Dysregulated pathways detection algorithm in a constructed gene network

CFinder is a clustering algorithm for detecting overlapping dense subgraphs based on the clique percolation method in the network [16]. This algorithm has been widely used in social networks, biological networks and microarray data to explore the module evolution process from a quantitative perspective. CFinder clustering algorithm provides an efficient clustering algorithm for large‐scale and relatively sparse network data, and has been widely applied to detect communities in social networks, and to detect the functional modules in biological networks [16].

Our detection algorithm works in the following two steps. In the first step, we utilise CFinder algorithm to obtain all the cliques in which mutations of all genes are mutually exclusive or they have interrelationships between gene mutations and gene expression changes in the gene network constructed in the previous step. In the second step, we calculate the overlap score between any two cliques using formula (4), identify the two gene sets with the highest overlap score, and then merge the two gene sets if their overlap score is higher than 0.25, which means that the intersection is at least half of the clique size if the two cliques are equal in size. The process repeats until there are no two gene sets whose overlap score is >0.25

(4) $$\text{OS}\left(A,B\right)=\frac{{\left|A\cap B\right|}^{2}}{\left|A\right|\left|B\right|}$$

where *A* and *B* stand for two gene sets in two cliques.

### 2.5 Parameter settings

In this algorithm, thresholds λ and γ are commonly applied to decide whether there is an edge between each pair of genes. λ is applied to describe the exclusive degree of a pair of genes and γ is applied to describe the ratio between non‐overlap and coverage of a pair of genes. If λ=1 and γ=1, it is an ideal case for a pair of genes to be mutually exclusive, whereas there often exist noises which may disrupt the exclusivity in a real mutation data [3, 6], so λ and γ are usually smaller than 1. In the process of constructing a gene network, λ=0.95 is chosen in line with previously reported in [6].

Formula (3) is introduced to avoid the case where two genes have a high exclusive degree but a low weight degree. We analyse different weight degrees of two genes with the same coverage, coverage overlap and exclusive degree in Fig. 2. The numbers in the figure represent coverage in different cases. According to the above formulas, we calculate coverage, coverage overlap and exclusive degree of the two genes in the four cases, which are 95, 5 and 0.95, respectively. However, the weight degrees are different in the four cases: (a) $\text{WD}(g_{j},g_{k})=0.9$, (b) $\text{WD}(g_{j},g_{k})=0.8$, (c) $\text{WD}(g_{j},g_{k})=0.67$, (d) $\text{WD}(g_{j},g_{k})=0$. Although the coverage, coverage overlap and exclusive degree of the two genes in the four cases are the same, the two genes in Figs. 2*a* and *b* are usually regarded as exclusive, while the two genes in Figs. 2*c* and *d* are not regarded as exclusive [10]. Here we choose γ=0.8 in the algorithm, as the experiment shows this produce an ideal result.

## 3 Results

To verify the validity of this method, we apply it to real gene mutations and gene expression data of glioblastoma, and detect two important dysregulated pathways of glioblastoma. Also, many genes in the two dysregulated pathways are found to have a strong correlation with glioblastoma.

### 3.1 Gene mutations and expression data of glioblastoma

The experimental data are derived from the gene mutations and expression data of glioblastoma in TCGA [17]. Gene mutations include single‐nucleotide variation and DNA copy number variation. The mutation data sets consist of 362 samples and 1, 8009 genes in which five genes, TTN, MUC16, PETN, TP53 and epidermal growth factor receptor (EGFR), in 126, 109, 107, 102 and 94 samples, respectively, mutate in more than 90 samples. A total of 334 patient samples and 9777 genes are selected from the data consisting of both gene mutations and expression. The genes which mutate in a small number of patients usually have no effect on cancer, and they are regarded as passenger mutations [7]. Therefore, we choose the genes whose mutation rates are higher than 3%, and get a total of 912 genes. Table 1 presents the details of the mutation data, including the number of samples, the number of genes, the average number of mutated gene per sample, and the average number of mutated sample per gene for the following two cases.

**Table 1:** Glioblastoma multiforme data used in this study

Gene selection	#patient	#gene	AMG	AMS
all genes	334	9777	96.29	3.29
mutation rates ≥3 percent	334	912	46.45	16.99

#patient: the number of patients. #gene: the number of genes. AMG: average number of mutated genes per sample. AMS: average number of mutated samples per gene.

### 3.2 Analysis of experimental results

Glioblastoma is the most aggressive cancer that begins within the brain, and it is a common and highly invasive primary central nervous system tumour generated from the neuroepithelial cells, accounting for about half of all intracranial primary tumours. The molecular alteration of primary glioblastoma is mainly due to the amplification and overexpression of EGFR, while the molecular alteration of secondary glioblastoma is predominantly due to the mutation of TP53 [18–20].

**Fig. 3:**
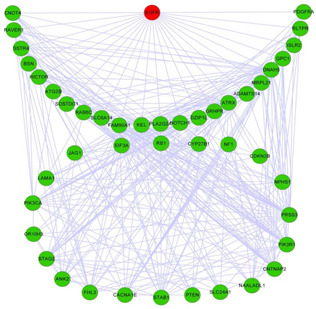
Dysregulated pathway revolving around EGFR

Based on the gene interaction network constructed by integrating gene mutations and expression data of glioblastoma, we firstly utilise CFinder algorithm to find all the cliques, and then merge two gene sets if their overlap score is >0.25. Finally, we obtain two dysregulated pathways, in which the first group is composed of 45 genes revolving around the EGFR shown in Fig. 3. There are 23 genes which directly interact with EGFR in the dysregulated pathway. The *P*‐values of the dysregulated pathway are $7.9\times 10^{-6}$ in melanoma, $2.3\times 10^{-4}$ in cancer pathway, $9.3\times 10^{-4}$ in the cell cycle pathway, and $2.2\times 10^{-4}$ in MAPK signalling pathway, according to DAVID functional annotation tool (http://david.abcc.ncifcrf.gov/summary.jsp). The *P*‐values shows that the dysregulated pathway is enriched in the above signalling pathways and is likely to be closely related to primary glioblastoma [18].

Among the 45 genes in this predicted dysregulated pathway, there are 23 genes reported to be related to glioblastoma. Table 2 lists the 23 genes and PubMed IDs of the papers that report the relationship between the genes and glioblastoma. Especially, EGFR and PTEN in the dysregulated pathway identified in our study are reported to be associated with glioblastoma in more than 100 previous studies.

**Table 2:** Genes related to glioblastoma in the predicted dysregulated pathway in literature with corresponding PUBMED IDs [21]

Genes	PMID
EGFR	27477273; 27450763; 27379987; 27303300; 27286795; 27167112 (top 6 among over 100)
PIK3R1	23166678; 22064833
NF1	23108917; 22943956; 21931722; 20405509; 20129251; 20029672 (top 6 among over 10)
PIK3CA	26902608; 25982275; 24469053; 22064833; 22026810; 17235514 (top 6 among over 10)
PTEN	27391443; 27292259; 27261630; 27239959; 27210502; 27073544 (top 6 among over 100)
RB1	27344175; 22157621; 14519639; 11204276; 8286200
CDKN2B	26839018; 19578366; 10541865
CYP27B1	16061850; 12899520; 11309335
PDGFRA	26320507; 23438035; 23074200; 22479456; 22323597; 21393858 (top 6 among over 10)
ATRX	27478330; 27314101; 26936505; 25479829; 25427834; 23104868
NOTCH1	26916895; 26662803; 26165719; 24898819; 23349727; 22249262 (top 6 among over 10)
SSTR4	9440032
RICTOR	27239959;23555046; 21557327
ATG2B	24792437
BSN	26701969
ISLR2	26934681
GPC1	24019070
FHL3	25659096
STAG2	24356817; 24088605; 21852505
EIF3A	22234522
JAG1	26546995; 22296176
LAMA1	18398573
STAB1	22960114

**Fig. 4:**
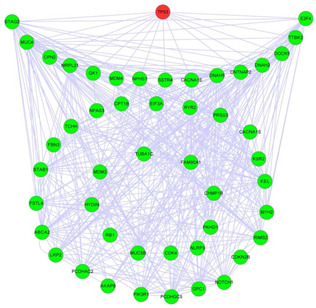
Dysregulated pathway revolving around TP53

The second group is composed of 50 genes revolving around TP53 mutation shown in Fig. 4, and there are 15 genes directly interacting with TP53. The *P*‐values of the dysregulated pathway are $1.1\times 10^{-6}$ in the cell cycle pathway, $8.8\times 10^{-6}$ in glioblastoma, $1.6\times 10^{-5}$ in melanoma, $2.5\times 10^{-4}$ in the p53 signalling pathway, $7.5\times 10^{-4}$ in the feedback loop pathway and $3.4\times 10^{-3}$ in the cancer pathway according to the DAVID functional annotation tool. This dysregulated pathway is enriched in these pathways and is likely to be closely related to secondary glioblastoma [19].

Among the 50 genes in this predicted dysregulated pathway, there are 19 genes reported to be related to glioblastoma. Table 3 lists the 19 genes and PubMed IDs of the papers that report the relationship between the genes and the glioblastoma. Especially, MDM2 in our identified dysregulated pathway is reported to be associated with glioblastoma in more than 100 previous studies.

**Table 3:** Genes that are reported to be related to glioblastoma in the predicted dysregulated pathway in literature with corresponding PUBMED IDs [21]

Genes	PMID
TP53	27478330; 26553592; 26482041; 26469958; 26258493; 25994230 (top 6 among over 50)
NOTCH1	26916895;26662803;26165719;24898819;23349727;22249262 (top 6 among over 10)
MDM2	27177180;27050782;26761214; 26482041; 26428461; 26328271 (top 6 among over 10)
MDM4	26328271; 24445145
CDKN2A	26839018; 23311918; 22046342; 21987724; 19086579; 12175345 (top 6 among over 10)
CDK4	27370397; 26649278; 26328271; 26149830; 23761023; 23707559 (top 6 among over 20)
RB1	27344175; 22157621; 21397855; 14519639; 11204276; 8286200
CDKN2B	26839018; 19578366; 10541865
PIK3R1	23166678; 22064833; 19305146; 14655756; 15605984
CPT1B	24618825
SSTR4	9440032
E2F4	10766737
MUC4	24582898
STAG2	24356817; 24088605
RIMS2	14997935
ABCA2	17415208
EIF3A	22234522
GPC1	24019070
NLRP3	25628952

## 4 Comparison with other methods

In order to validate our algorithm, we analyse the genes in the dysregulated pathways identified by this algorithm with the literature corresponding PUBMED IDs in which the genes are reported to be related to glioblastoma. It is necessary to point out although a number of methods have been proposed to identify dysregulated pathways or driver pathways in cancer, including a method based on a pathway interaction network [2], integrative enrichment analysis method [4], multi‐Dendrix [5], network‐based method (NBM) [6], Dendrix [7], gene interaction enrichment and network analysis method [8], MDPFinder [10], CONTOUR[11], MEMCover [12] and a method based on statistical models (MBSM) [3], none of the above utilised the mutually exclusive relationship between genes and the interrelationship between gene mutations and gene expression changes to identify dysregulated pathways in cancer. Here we compare our method against MBSM [3] to analyse the differences between the two data integration methods, and compare our method against NBM [6] to analyse the differences between results of the two methods in glioblastoma data.

MBSM [3] proposed a linear modelling approach to combine gene mutations with expression data for studying genetic mechanisms of mutation‐induced expression changes, prediction of blood counts, and prognostic power of mutations, expression and clinical data. The method analysed the relationships between mutations in 12 genes which frequently mutate in the myelodysplastic syndromes for 124 patients. The study found that the number of differentially expressed genes vary extensively among driver alterations, and both genotype and expression data have a similar contribution to blood counts.

NBM [6] proposed a network‐based method to detect overlapping driver pathways automatically only utilising somatic mutation data in cancer. The method presented the exclusive degree function $\text{ED}(g_{i},g_{j})$ of the two genes rather than the weight degree function $\text{WD}(g_{i},g_{j})$. According to their method, the four cases in Fig. 2 were regarded as mutual exclusive gene pair, whereas the cases of Figs. 2*c* and *d* are usually not regarded as a mutual exclusive gene pair (as can be seen from Fig. 2). In our algorithm, the weight degree function defined can eliminate the cases of Figs. 2*c* and *d*, which thus can identify the real exclusive gene pairs.

To validate the improvement of the proposed algorithm, we compare the results of proposed algorithm and NBM by applying them on the mutation dataset (see Table 4). As can be seen, NBM detects six driver pathways (S1, S2, S3, S4, S5 and S6) based on the default parameters (λ=0.95 and δ=0.3) [6]. In our method, threshold λ of the exclusive degree is set to be 0.95 and threshold γ of the weight degree is set to be 0.8 based on the performance of training samples. Running on the same mutation data, our method detects two dysregulated pathways (Set1 and Set2). The first dysregulated pathway (Set1) detected by our algorithm contains the genes in three driver pathways (S1, S2 and S3) detected by NBM completely, and the second dysregulated pathway (Set2) encompasses the genes in three driver pathways (S4, S5 and S6) detected by NBM. This indicates that the three gene sets (S1, S2 and S3) are the driver pathways of the primary subtype of glioblastoma, and the three gene sets (S4, S5 and S6) are driver pathways of the secondary subtype of glioblastoma. Also, different combinations of mutations in the three gene sets (S1, S2 and S3) perturb the Set1 dysregulated pathway, and then lead to the primary subtype of glioblastoma, and different combinations of mutations in the three gene sets (S4, S5 and S6) perturb the Set2 dysregulated pathway, and then lead to the secondary subtype of glioblastoma.

**Table 4:** Comparison between the results of NBM and our method in Glioblastoma Multiforme data

Results of NBM		Results of our method	
S1	EGFR, PIK3CA PTEN, NOTCH1	Set1	EGFR, PIK3R1, NF1, PIK3CA, PTEN, RB1, CDKN2B, CYP27B1, PDGFRA, ATRX, NOTCH1, SSTR4, RICTOR, ATG2B, BSN, ISLR2, GPC1, FHL3, STAG2, EIF3A, JAG1, LAMA1, STAB1
S2	PTEN, PIK3R1, PIK3CA, STAG2		
S3	EGFR, PK3R1, NF1, CYP27B1, STAB1		
S4	TP53, CDKN2B, CDKN2A, RB1, CDK4	Set2	TP53, NOTCH1, MDM2, MDM4, CDKN2A, CDK4, RB1, CDKN2B, PIK3R1, CPT1B, SSTR4, E2F4, MUC4, STAG2, RIMS2, ABCA2, EIF3A, GPC1, NLRP3
S5	TP53, MDM2, MDM4, QK1		
S6	TP53, PIK3R1, MDM2, CPT1B		

## 5 Conclusions

In this study, we construct a gene network by using the interrelationship between gene mutations and expression changes, and using the mutually exclusive relationship in genome spectrum, and then using CFinder clustering algorithm to identify cliques in the constructed gene network. We apply the algorithm to gene mutations and gene expression data of glioblastoma, and identify two dysregulated pathways, which are closely related to the two subtypes of glioblastoma. The results show that one dysregulated pathway revolving around EGFR is likely to be associated with the primary subtype of glioblastoma, and the other dysregulated pathway revolving around TP53 is likely to be associated with the secondary subtype of glioblastoma. Most genes in the two identified dysregulated pathways have been previously reported to be directly related to glioblastoma. Especially, 23 of the 45 genes in the first dysregulated pathway are directly related to glioblastoma, and 19 of the 50 genes in the second dysregulated pathway are directly related to glioblastoma. This algorithm provides a useful complement to the correlation between gene mutations and gene expression in cancer, and enriches our understanding of the molecular pathogenesis of cancer. The algorithm also provides a supplement to the analyses of cancer data and a useful basis for dysregulated pathways detection.
